# Genetics of Behçet's Disease

**DOI:** 10.1155/2012/912589

**Published:** 2011-10-16

**Authors:** Tamer İrfan Kaya

**Affiliations:** Department of Dermatology, Faculty of Medicine, Mersin University, 33079 Mersin, Turkey

## Abstract

Behçet's disease (BD) is a systemic inflammatory disorder characterized mainly by recurrent oral and genital ulcers and eye involvement. Although the pathogenesis remains poorly understood, a variety of studies have demonstrated that genetic predisposition is a major factor in disease susceptibility. Peculiar geographical distribution of BD along the ancient Silk Road has been regarded as evidence supporting genetic influence. The observed aggregation of BD in families of patients with BD is also supportive for a genetic component in its etiology. HLA-B51 (B510101 subtype) is the most strongly associated genetic marker for BD in countries on the Silk Road. In recent years, several genome-wide association studies and genetic polymorphism studies have also found new genetic associations with BD, which may have a supplementary role in disease susceptibility and/or severity. The author reviewed the HLA and non-HLA genetic association studies.

## 1. Introduction

Behçet's disease (BD) is a complex syndrome characterized mainly by recurrent oral aphthous ulcers, genital ulcerations, and ocular involvement. This triple-symptom complex was first described by Hulusi Behçet, a Turkish dermatologist, as a separate disease entity. Later, other associated clinical features were described [1, 2]. Although the aetiology is still obscure, BD is believed to be triggered by environmental factors such as microbial agents in individuals with a particular genetic background. Interactions of genetic and environmental factors in BD patients may underlie pathogenic processes, which may influence the development of the disease and modify its course. The prevalent distribution in a specific geographical area, the close association with HLA-B51 in different ethnic groups, and the familial clustering of BD are hallmarks accounting for the strong contribution of a genetic background [2, 3]. Here we review the current knowledge of the genetic basis of BD and their implications in pathogenesis of BD and summarize the important findings in Table 1.

## 2. Geographical Distribution

The prevalence of BD is known to be high in Japan, China, Turkey, and the Mediterranean and the Middle Eastern countries. It is hypothesized that this geographical distribution of BD from Japan to the Middle East and the Mediterranean basin correlates with the distribution of HLA-B51 by old nomadic or Turkish tribes via the ancient Silk Road. This peculiar geographical distribution have been regarded as evidence supporting genetic influence on the pathogenesis of BD [1, 4, 5]. The highest prevalence rates of BD were reported from Turkey as 420/100.000 in Istanbul, 380/100.000 in a rural area in northern Turkey, and 110/100.000 in Ankara [6–8]. Usually prevalence rates between %7/100.000 and %30/100.000 were reported in Middle East, Mediterranean, and Far East countries [1, 9]. It is rarely seen in Western and Northern Europe and United States, for example, its prevalence reported 2,26/100.000 in Germany and 5,2/100.000 in the United States [10, 11]. In sub-Saharan Africa, Australasia, and among Amerindians the disease is rare or almost absent [9]. The prevalence of BD in Berlin was reported to be the highest among Lebanese (101.3/100 000) and Turkish (77.3/100 000) people, while the lowest prevalence was reported among Germans (1.4/100 000) [12]. These findings suggest that BD prevalence is mostly dependent on ethnic origin and genetic factors rather than environmental factors [9, 12].

## 3. HLA-B51 and Other HLA Genes Associated with BD

HLA-B51, one of the split antigens of HLA-B5, has been found to be the most accurate genetic marker for BD to date in different ethnic groups. However, its contribution to the overall genetic BD susceptibility was estimated about to be 20% [13]. The HLA-B51 is frequent in BD patients, with a range of 40–80% in ethnic groups including Turkish, European, and Asian populations from the Middle East to the Far East, whereas it can be as low as 13% among white patients in western countries [3, 9]. And in our study, we observed HLA-B51 in 46 (54.1%) of our 85 Turkish patients [1]. In general, the strongest correlation between BD and HLA-B51 is seen among populations with a high incidence of HLA-B51 and BD, and the weakest correlation is seen in populations where BD and HLA-B51 are rare [1, 9]. In a recent meta-analysis of 4,800 patients with BD and 16,289 controls from 78 independent studies, the pooled odds ratio (OR) of HLA-B51/B5 allele carriers to develop BD compared with noncarriers was found to be 5.78 (95% CI 5.00–6.67). They found that the pooled rates of HLA-B51/B5-positive BD cases varied across geographic locations, but interestingly the relative risk increases associated with this allele appeared to be fairly even for different ethnic populations [14].

There are more than 89 different subtypes of HLA-B51, and HLA-B5101 is the major suballele associated with BD in all the populations studied. HLA-B5108 was also found to be associated with BD in the Middle Eastern, Italian, Spanish, Greek, Turkish, and German patients. Oppositely, it has been suggested that HLA-B5107 might be negatively associated with BD in the Turkish and German populations [3, 9]. Recently, Y. Takemoto et al. investigated *HLA-B5101* gene from Japanese, Turkish, Jordanian, and Iranian patients and found that all the patients have B510101 suggesting that the susceptibility to BD was conferred by the B510101 subtype and not by any genes in linkage disequilibrium with HLA-B51 [15]. Accordingly, Amerindian populations, known to carry a high frequency of HLA-B51 but having no BD incidence, are known to carry the highest frequencies of nonsusceptible HLA-B510201 allele [9, 16]. 

In our study, we also investigated the association of HLA class I alleles with the manifestations of BD. We found that BD patients with HLA-B51 antigen are almost four times more likely to have thrombophlebitis than patients without it. We also found some less significant associations: decreased HLA-B35 frequency in patients with thrombophlebitis, increased HLA-A29 and decreased HLABw6 frequency in patients with ocular involvement, decreased HLA-Cw2 frequency in patients with erythema nodosum, and decreased HLA-Cw7 frequency in patients with genital ulceration [1]. Zouboulis et al. also found that both superficial thrombophlebitis and deep thrombosis significantly were more frequent in HLA-B5-positive BD patients compared with HLA-B5-negative patients. They did not observe any other association between the class I HLA antigens and manifestations of BD [17]. Alekberova et al. found superficial thrombophlebitis statistically more frequent in HLA-B5-positive BD patients [18]. However, Muftuoglu et al. observed no HLA allele association with thrombophlebitis, ocular involvement, arthritis, and erythema nodosum in BD patients [19].

The pathogenetic role of HLA-B51 in BD has yet to be elucidated. However, HLA-B51 molecule itself may be responsible, at least in part, for neutrophil hyperfunction in BD, since HLA-B51-transgenic mice show enhanced neutrophil function as seen in BD patients, although these mice did not develop the symptoms of BD [20]. Because a primary role of HLA class I antigens such as HLA-B51 is to present endogenous peptides to CD8+ T cells, the lack of the disease phenotype in this mouse model can be explained by the absence of an triggering microbial or injury-related peptide that would activate the disease-relevant CD8+ T cells. MICA (9-mer peptide AAAAAIFVI) is a stress-inducible antigenic peptide, and it is one of the many triggering candidates of BD. Yasuoka et al. showed that only HLA-B51-positive BD patients with active disease showed a MICA-mediated cytotoxicity and they also showed that this specific T cell response was lost after the BD-related symptoms disappeared [21]. Superoxide production by neutrophils was also reported to be increased in HLA-B51-positive individuals [22]. These findings suggest that HLA-B51 could be associated with neutrophil hyperfunction in BD.

Several other HLA class I and class II alleles including HLA-A26, HLA-B15, HLA-B5701, HLA-B2702, HLA-B3901, HLA-B52, HLA-B56, Cw1, Cw14, Cw15, Cw16, HLA-DRB104, and HLA-DRB107 have been described to be associated with BD in different populations. Positive and negative associations with nonclassical HLA-E, HLA-F, and HLA-G polymorphisms were also detected in Korean and Japanese patients. But because of their low incidence or the small size of investigated populations, mostly significance levels have been weak and most of them were achieved in single studies only. Therefore, they are generally not considered to give primary susceptibility to the disease. In addition, since there is strong linkage disequilibrium within the MHC region it is not clear whether these associations cause susceptibility to BD or they have a linkage disequilibrium with HLA-B51. Among these associations HLA-A26, HLA-B15, and HLA-B5701 were found to be independently associated with BD [1, 3, 9, 23–25]. 

The *MHC class I chain-related gene A (MIC-A)* was also regarded as a candidate for BD genetic susceptibility. Several studies have demonstrated associations between MIC-A009, MIC-A006, MIC-A6 TM, and BD. However, these associations appear to be the result of a strong linkage disequilibrium of MIC-A with HLA-B51, so today they are not considered as the primary susceptibility genes for BD [9, 26].

## 4. Genome-Wide Association Studies (GWASs)

GWAS can facilitate new unbiased biologic insights into disease pathogenesis, and they can provide more definite answers for the cause of the diseases of complex genetic trait when performed in large groups and confirmed in different populations. The first GWAS was relatively small and was reported in Turkish BD patients: Fei et al. identified genetic associations between BD and single-nucleotide polymorphisms (SNPs) in KIAA1529, CPVL, LOC100129342, UBASH3B, and UBAC2 (OR = 2.04, 2.26, 1.84, 1.71, and 1.61, resp.). Interestingly, none of these five susceptibility loci had been associated with BD before. The functions of two are not known, and the genes *UBASH3B* and *UBAC2* both contain a UBA, suggesting that both gene products are involved in the ubiquitination pathway. *CPVL* gene encodes for a carboxypeptidase that cleaves a single amino acid from the carboxy terminus of proteins or peptides [27].

Remmers et al. performed a GWAS with 311,459 SNPs in 1215 BD patients and 1278 healthy controls from Turkey. They confirmed HLA-B51 association and identified a second, independent association within the MHC Class I region. In addition, the results of the meta-analysis including a total of 2430 cases and 2660 controls established associations with the interleukin10 (IL10) variant (OR: 1.45) and with a variant located between the *IL23 receptor* (IL23R) and *IL12 receptor *β*2* (IL12RB2) genes (OR: 1.28) [28]. Recently, Mizuki et al. performed a similar GWAS using a larger sample pool using 500, 568 SNPs in 612 Japanese individuals with BD and 740 unaffected controls, and the HLA-B region showed the most significant association with BD (rs4959053, *P* = 1.8 × 10^−26^). They also detected genome-wide significant associations outside the HLA complex, at *P* = 2.7 × 10^−8^ for rs12119179, which is located in the 47 kb intergenic region between IL23R and IL12RB2. The second strongest region of association they found was rs1554286, located within the intron 3 of IL10 (*P* = 8.0 × 10^−8^) [29]. The results of these 2 large GWASs provide cross-validation of these two non-HLA regions, IL10 and IL23R-IL12RB2 associations in two distinct populations. IL23 is a heterodimeric pro-inflammatory cytokine that has been shown to stimulate T helper cell proliferation and increase the production of inflammatory cytokines such as IL1, IL6, IL17, and TNF*α*. IL10 is a potent suppressor of inflammatory cytokines such as IL1, IL6, IL12, TNF*α* and IFN*γ* and inhibits T cell and NK cell activation [29].

Meguro et al. also performed a GWAS in BD among 23.465 microsatellites, and 6 best positively associated microsatellites (of a total of 147) (D3S0186i, D6S0014i, D6S0032i, 536G12A, D12S0645i, and D22S0104i) with BD were identified. New studies are needed to identify the importance of these microsatellites in the pathogenesis of BD. They also performed a class I HLA analysis. They found HLA-B5101 as the strongest susceptibility allele for the development of BD. Interestingly, among the HLA-B5101-negative patients, HLA-A26 was found to be the most strongly associated allele with BD (allele: 25.9% versus 10.6%, OR = 2.96). Significantly increased incidences of HLA-F010101 and HLAG010102 in patients with BD probably result from linkage disequilibrium with HLA-A26 [30]. In previous studies, HLA-A26 was significantly associated with BD in Japanese, Taiwanese, and Greek populations. On the contrary, in Ireland, Italy, and Turkey, HLA-A26 was increased in healthy controls compared with patients with BD, but not significantly [30]. Karasneh et al. performed a whole-genome linkage analysis with 395 microsatellite markers and identified 16 potential loci for BD, 1p36, 4p15, 5q12, 5q23, 6p22–24, 6q16, 6q25-26, 7p21, 10q24, 12p12-13, 12q13, 16q12, 16q21–23, 17p13, 20q12-13, and Xq26–28 with the strongest evidence seen for 12p12-13 and 6p22–24 [31]. Two chromosome regions were consistent with the regions of two BD susceptibility markers, 536G12A and D12S0645i, which were located on 6q25.1 and 12p12.1, which were found by Meguro et al. in their genome-wide association study [30].

## 5. Familial Aggregation

Familial aggregation has been also regarded as an evidence supporting the genetic predisposition to BD and has been reported in 1–18% of the patients, mostly of Turkish, Israeli and Korean origin, and it is increased in patients with juvenile disease and is especially manifest in families of probands carrying HLA B51. The familial aggregation of BD has been observed in different ethnic groups with a variable frequency. It is higher in Turks (18.2%), Koreans (15.4%), and Jews (13.2%) than in Chinese (2.6%), Japanese (2.2%), and Europeans (1%) [3, 32–34]. 

Analysis of a small group of multicase families did not demonstrate any particular Mendelian inheritance pattern [35]. However, a recent study provided evidence of an autosomal-recessive Mendelian mode of inheritance in a pediatric BD subgroup suggesting that genetic load might be higher in children with BD than in adults [36]. A genetic anticipation, in the form of earlier disease onset in the children of the affected parents, has also been observed in a group of Turkish families. The expansion of unstable trinucleotide repeats has been proposed as the genetic basis of the defect in familial cases [37]. Treudler et al. observed that juvenile-onset disease was characterized by an increase in familial cases (25% versus 8% in patients with adult onset; *P* = .047) [38]. Laghmari et al. evaluated clinical characteristics of BD in Moroccan children, and they reported the familial disease in 30.7% of their cases, which is the highest familial aggregation rate reported so far [39]. I. Koné-Paut reported that 13 of the 106 pediatric patients (12.3%) and 9 of the 399 nonpediatric patients (2.2%) had relatives affected by BD. This excess of familial cases in the pediatric group compared with the nonpediatric group was found to be significant (*P* < .0001). They also found that the mean age of attaining criteria in familial cases (17. 95 years) was significantly lower than in sporadic cases (27.28 years; *P* < .0001), and they proposed the inclusion of familial history in the definition of pediatric BD [40].

Akpolat et al. evaluated 27 patients with familial BD in 12 families. Among the 137 patients they studied, the rate of familial form of BD was 8.7%. Vascular involvement was 7.4% (2/27) in the familial group, while it was 28.8% (36/125) in patients without the familial form of the disease (*P* < 0.01). They also observed a high rate of (68%) HLA-B51 positivity in the familial form [33]. Recurrence most commonly occurs between siblings, but also there are mother/son, mother/daughter, father/son, father/daughter, cousin/cousin and uncle/nephew cases [3]. The sibling recurrence risk of BD was reported to be 4.2% in Turkey, and the recurrence risk ratio (*λ*s) was estimated to be 11.4–52.5 in Turkish populations [41]. In familial BD, the HLA-B51 positivity is much more frequent than in sporadic cases, being 68-83.3% in Turkish studies. The HLA-B51 homozygosity is common in familial cases [33, 42]. 

Twin concordance studies are frequently being used for estimating the role of genetic factors in the pathogenesis of multifactorial diseases. In a recent twin study of Masatlioglu et al., the pairwise concordance rate for BD was 2/6 for monozygotic twins and 1/8 for dizygotic twins. In this study genetic effects accounted for 41% of the phenotypic variance for BD among twins. The higher concordances for BD in monozygotic twins compared with dizygotic twins suggests genetic predisposition [43]. In addition, there are two reports of monozygotic twins concordant for BD [43, 44]. In one of these twins reports both patients interestingly showed intestinal BD; however, they were HLA-B51 negative [44]. In the other report, the twin brothers were both HLA-B51 positive and developed the disease at the same age [45]. There is also one report of a pair of HLA-B51-negative monozygotic twins discordant for BD in the literature [46].

## 6. Immunogenetics and Gene Polymorphism Studies

Cytokine-mediated immunity plays a crucial role in the pathogenesis of various immunologically mediated diseases. It has been suggested that polymorphisms of genes involved in immunopathogenesis of diseases combining with environmental factors may be important in the development of these diseases. Several genes such as *IL* genes (*IL-1A, IL-1B, IL-1 receptor antagonist, IL-2, IL-6, IL-10, IL-12B promoter, IL-17F, IL-18, IL-23R* genes), *tumor necrosis factor (TNF)* genes, *transporter associated with antigen processing (TAP)* gene, *intercellular adhesion molecule-1 (ICAM-1)* gene, *endothelial nitric oxide synthase* (eNOS) gene, *glutathione S-transferase* gene, *N-acetyltransferase* gene, and *vascular endothelial growth factor (VEGF)* gene polymorphisms have been related to BD susceptibility. However, in these studies significance levels were mostly weak, most of them were achieved in single studies and if there are multiple studies the results are mostly conflicting. For example, *eNOS* gene polymorphism was reported to be associated with BD susceptibility in Italian and Korean, but not in Turkish and Japanese patients. Therefore their role in BD pathogenesis is not clear, and further investigations are needed for definitive conclusions [3, 47–52]. 

Thrombosis is a common complication of BD and the pathogenic mechanism of thrombotic tendency in BD is not well known. The role of procoagulant polymorphisms and mutations has also been investigated in BD. As *platelet membrane glycoprotein* gene polymorphisms have been identified as risk factors for thrombosis we investigated the association of the *platelet glycoprotein Ia C807T/G873A* gene polymorphism and thrombosis in BD and we found that the risk of thrombosis is significantly higher in patients who have 807TT and 807CT genotypes than in patients who have 807CC genotype [53]. Associations of factor V Leiden and *prothrombin* gene mutations with BD were confirmed in some studies, but not in others [3, 54, 55]. We also investigated the association between the factor V Leiden and *prothrombin* gene mutations with BD and we found a significantly high prevalence of the *prothrombin* gene mutation in patients with BD patients than in control subjects [56]. 

Here we review the current knowledge of the genetic basis of BD. However, it has been evident that there are similarities and differences in BD susceptibility loci across ethnic groups. Therefore, efforts to identify ethnic-specific genetic factors or disease-causing variants are needed for the genetic dissection of BD.

## Figures and Tables

**Table 1:** Findings supporting the genetic background of Behçet's disease.

Studies	Findings/associations
Epidemiology	Geographical distribution: from Japan to the Middle East and the Mediterranean basin

Ethnic origin	Prevalence in Berlin: high in Lebanese and Turks and low in Germans

HLA	HLA-B51 (B510101 subtype)

Genome-wide association studies	(i) MHC Class I region: HLA-B51 (B5101)
	(ii) Non-HLA regions: IL10 region and region between IL23R and IL12RB2
	(iii) Chromosome regions: 6q25-26, 12p12–13

Familial aggregation	(i) In patients with pediatric onset
	(ii) Autosomal-recessive Mendelian mode of inheritance in a pediatric subgroup

Twin concordance studies	High concordances in monozygotic twins

Gene polymorphism studies	Inconsistent and weak associations, further investigations are needed for definitive conclusions
